# TSH levels after fresh embryo transfer are associated with reproductive outcomes in euthyroid women undergoing the first IVF/ICSI cycles

**DOI:** 10.1038/s41598-023-36276-0

**Published:** 2023-06-02

**Authors:** Yuchao Zhang, Yanli Liu, Wenbin Wu, Zhen Li, Bingnan Ren, Yichun Guan

**Affiliations:** Department of Reproductive Medicine, The Third Affiliated Hospital of Zhengzhou University, No.7 Kangfuqian Street, Erqi, Zhengzhou, 450052 Henan China

**Keywords:** Endocrinology, Health care

## Abstract

To investigate whether there is a relationship between TSH levels on the 14th day post embryo transfer (D14 TSH levels) and the reproductive outcomes in euthyroid women who are free from levothyroxine (LT4) treatment and undergo the first in vitro fertilization /intracytoplasmic sperm injection embryo transfer (IVF/ICSI-ET) cycles with the homogeneous ovarian stimulation protocols. This was a prospective study including a total of 599 euthyroid women undergoing the first IVF/ICSI ET cycles. Serum samples were collected and frozen on the 14th day post embryo transfer. TSH levels were measured after the confirmation of clinical pregnancy. The patients were divided into three groups (low-normal ≤ 2.5 mIU/L; high-normal 2.5–4.2 mIU/L; and high > 4.2 mIU/L) based on D14 TSH levels. Reproductive outcomes were compared among the three groups. Binary logistic regression analyses and generalized additive mixed models with smoothing splines were used to investigate the relationship between TSH levels and reproductive outcomes. D14 TSH levels were significantly elevated compared to basal TSH levels, and the degree of TSH elevation was significantly higher in pregnant women compared to that in non-pregnant women. The clinical pregnancy and live birth rates increased significantly in the high-normal D14 TSH groups, and doubled in the high D14 TSH groups compared to the low TSH groups. When adjusted by age, basal TSH, AMH, E2, endometrial thickness, type and causes of infertility, and transferred embryos, the dose-dependent relationships between D14 TSH and clinical pregnancy and live birth were observed. Obstetric outcomes in singleton or twins live birth among the different D14 TSH groups were similar. Elevated D14 TSH levels were associated with better clinical pregnancy and live birth rates, and were not associated with worse obstetric outcomes. The mechanisms to explain the phenomenon remained to be studied.

## Introduction

Via the hypothalamic-pituitary-thyroid axis, thyroid-stimulating hormone (TSH) regulates the synthesis and secretion of thyroid hormones (TH), which play a vital role in the mechanisms regulating implantation and early fetal development^1^. However, it is well accepted that controlled ovarian stimulation (COS), which is characterized by superphysiological estrogen levels, could affect the TH and TSH levels via the alteration of serum thyroid binding globulin (TBG)^2^. Previous studies extensively investigated the modifications of thyroid function during in vitro fertilization/intracytoplasmic sperm injection (IVF/ICSI) cycles, and claimed significant elevation of TSH levels^3–9^. According to the previous studies, it should be noted that TSH levels during the COS may not truly reflect the function of thyroid gland due to the effects of super-physiological estrogen levels and exogenous recombinant human chorionic gonadotropin (rHCG).

The data about the associations between TSH levels on the pregnancy test day and early reproductive and long term follow-up outcomes are limited^4,9–11^. Generally, women are often required to visit the hospital for pregnancy test on the 14th days post embryo transfer (ET), when the effect of drug-induced change of TSH declines and physiological TSH change induced by embryonic HCG appears. It was proposed that impaired thyroid response to HCG may led to implantation failure or miscarriage^1^. However, what is the normal thyroid response should be defined properly. Previous studies indicated that the normal thyroid response was the condition that high HCG levels was positively associated with TH levels and negatively with TSH levels^12^. However, the gestational weeks in the previous studies were quite different from the 2 weeks for the women with fresh ET, which indicated that the normal thyroid response may be a different condition.

Moreover, few studies investigated the impact of D14 TSH levels on the reproductive outcomes, especially the obstetric outcomes^3–6,9–11^. We previously reported the association between TSH levels on the 14th day post ET (D14 TSH) and clinical outcomes in women who underwent frozen-thawed ET^13^, during which course the estrogen levels were apparently lower than those from the fresh ET cycles. In order to better understanding the role of D14 TSH levels in women who are offered with fresh ET, the aim of the study is to investigate whether there is relationship between D14 TSH levels and the reproductive outcomes in euthyroid women who are free from LT4 treatment and will undergo the first IVF/ICSI ET cycles with the homogeneous COS protocols.

## Material and methods

### Patients and groups

This was a prospective study which was approved by the Institutional Review Board of the Third Affiliated Hospital of Zhengzhou University. Infertile women having undergone IVF/ICSI treatment from October 2020 to June 2021 at the Department of Reproductive Medicine were initially included. Patients with the following criteria were initially included. (1) The patients underwent the first treatment cycle and had thyroid function screened. (2) The embryos were transferred freshly without vitrification. (3) The patients came back to the hospital on the 14th day post ET for pregnancy test. Here are the exclusive criteria: (1) mild ovarian stimulation protocol and gonadotrophin-releasing hormone antagonist (GnRH-ant) stimulation protocol; (2) overt or subclinical thyroid dysfunction before COS; (3) and treatment for thyroid dysfunction during pregnancy. Age, BMI, and AMH were not restricted in this study.

Infertile women who firstly visited the department for reproductive consultation were generally required to have thyroid function screened. When the thyroid parameters were confirmed normal, the TSH levels were recorded as baseline data. Otherwise, the patients were treated accordingly and excluded from this study. After treatment with COS and fresh ET, the patients would be advised to visit the department on the 14th day post ET for pregnancy test. The used serum was then frozen at − 20 ℃ for batch analysis at least one month after ET when the result of clinical pregnancy came out. It was anticipated that TSH levels of some patients on the pregnancy test day would rise beyond the upper limit of the reference range. Accordingly, the patients would be divided into three groups based on the D14 TSH levels (low-normal group, ≤ 2.5 mIU/L; high-normal group, 2.5–4.2 mIU/L; and high group, > 4.2 mIU/L).

### The process of COS and ET

The infertile women were supplied with GnRH-agonist (GnRH-a) protocols with a pituitary down-regulation according to their individual conditions. Detailed procedures were described elsewhere^14^. After oocytes pick-up, insemination with conventional IVF or ICSI method was performed. For those with IVF methods, granulosa cells of no more than 6 oocytes were removed for observation of 2 polar bodies, which were the signs of normal fertilization. When no more than 2 oocytes showed 2 polar bodies, rescued ICSI was performed for all mature oocytes to avoid failure of fertilization. The inseminated zygotes were cultured and observed each day until the third day when the decision of transferring single or two cleavage embryos was made, or the fifth day for single blastocyst transfer. The embryos of the top quality at the time being were chosen for transfer. Scheme of luteal-phase support was provided as described elsewhere.

### Laboratory analysis

The basal TSH levels were tested on the 2nd–4th day of the menstrual cycle, along with the measurement of basal sex hormones. The frozen serum samples were rewarmed at the room temperature for at least half an hour before being tested for TSH levels. All The measurements were performed by electrochemical luminescence (ECLIA) on a Cobas 8000 (Roche Diagnostics, Germany). The TSH reference range was provided by the manufacturer (TSH: 0.27–4.2 mIU/L). The range of intra-assay coefficient of variation (CV) of serum TSH was 1.39–2.59%, and the inter-assay CV 2.33–4.20%. Daily internal quality control and yearly external quality control were carried out by request.

### Definition of reproductive outcomes

The primary outcomes were clinical pregnancy rate defined as the presence of gestational sac and fetal heart activity per ET cycle, miscarriage rate defined as per clinical pregnancy that did not result in delivery, and live birth rate defined as delivery of live babies per ET cycle. Considering that dramatic differences existed between single and twin pregnancies, obstetrics outcomes were compared separately.

### Statistical analysis

Data were expressed as mean [standard deviation (SD)] or median [interquartile range (IQR)] for continuous variables, and as number (percentage) for categorical variables. Comparisons were performed by one-way ANOVA (normal distribution) or Kruskal–Wallis H-test (non-normal distribution) for continuous variables and chi-square analysis tests for categorical variables. TSH levels between the pregnancy test day and the baseline were compared using the paired t-test. Mean difference (MD) was calculated as the value between D14 TSH and basal TSH. The MD between pregnant and non-pregnant women was compared using independent t-test. In order to investigate the impact of grouped D14 TSH on the primary outcomes, such as clinical pregnancy, miscarriage, and live birth, binary logistic regression analyses were perform. Confounding factors such as female age, serum AMH levels, estrogen levels on the trigger day, and transferred embryos were included for adjustment. Comparisons among the different groups were performed by SPSS (Version 22.0 IBM; NY). The generalized additive mixed models with smoothing splines were employed to visually assess the relationship between D14 TSH and primary outcomes. If the non-linear correlation was observed, a two-piecewise linear regression model was performed to calculate the threshold effect of D14 TSH on primary outcomes in terms of the smoothing plot. The analyses were performed using Empower (R) (www.empowerstats.com, X&Y Solutions, Inc., Boston, MA), which is based on the statistical package R (The R Foundation; http://www.r-project.org; version 3.4.3). Significance was set at two-tailed *P* < 0.05 in all analyses.

### Ethics approval and consent to participate

The ethics committee of the Third Affiliated Hospital of Zhengzhou University approved this study. All methods were carried out in accordance with the Code of Ethics of the Declaration of Helsinki. The informed consent were obtained from the included study participants.

## Results

### General characteristics of included patients

A total of 599 euthyroid women undergoing the first IVF/ICSI ET cycles were included in this study, of whom 239 (39.9%) had low D14 TSH levels, 225 (37.6%) high-normal D14 TSH levels, and 135 (22.5%) high D14 TSH levels. As shown in Table 1, there were no significant differences in terms of most of general characteristics among the three groups. However, estrogen levels on the trigger day and serum AMH levels were significantly higher in the high TSH group (*P* = 0.004, respectively). The basal TSH levels significantly rose along with the D14 TSH levels (*P* < 0.001), while FT4 levels showed the opposite trend (*P* = 0.02). There were significant differences in terms of proportions of transferred cleavage embryos or blastocysts (*P* = 0.001) and number of good quality cleavage embryos (*P* = 0.003).

**Table 1:** General characteristics of included patients based on D14 TSH levels.

	Low (239)	High-normal (225)	High (135)	*P*
Age (years) mean (SD)	31.52 (4.15)	31.34 (4.57)	30.40 (4.50)	0.06
Endometrial thickness (mm) median (IQR)	11.00 (3.00)	11.00 (2.85)	11.50 (3.00)	0.10
AMH (pmol/L) median (IQR)	17.98 (13.39)	19.75 (13.39)	21.1 (13.27)	0.004
Infertility duration (years) median (IQR)	3.00 (3.50)	3.00 (3.00)	3.00 (3.00)	0.29
Basal TSH (mIU/L) mean (SD)	1.86 (0.75)	2.48 (0.76)	2.89 (0.75)	< 0.001
FT4 (pmol/L) mean (SD)	16.38 (2.19)	16.25 (2.13)	15.73 (2.03)	0.02
FT3 (pmol/L) mean (SD)	4.91 (0.61)	4.89 (0.58)	4.9 (0.59)	0.94
LH (IU/L) mean (SD)	5.56 (2.39)	5.63 (2.4)	5.86 (3.05)	0.54
FSH (IU/L) mean (SD)	6.91 (1.72)	6.91 (2.05)	6.8 (1.57)	0.84
E2 on the HCG day (pmol/L) median (IQR)	10,694.00 (6006.00)	9884.00 (5736.50)	11,795.00 (7532.00)	0.004
Type of infertility n (%)				
Primary	106 (44.35%)	107 (47.56%)	67 (49.63%)	0.59
Secondary	133 (55.65%)	118 (52.44%)	68 (50.37%)	
Causes of infertility n (%)				
Male factors	54 (22.59%)	46 (20.44%)	31 (22.96%)	0.99
Ovulation dysfunction	37 (15.48%)	31 (13.78%)	21 (15.56%)	
Tubal and pelvic factors	67 (28.03%)	63 (28.00%)	41 (30.37%)	
Female mixed factors	13 (5.43%)	12 (5.33%)	6 (4.44%)	
Male and Female factors	44 (18.41%)	50 (22.22%)	25 (18.52%)	
Unexplained	24 (10.04%)	23 (10.22%)	11 (8.15%)	
Transferred embryos n (%)				
Cleavage embryos	160 (66.95%)	160 (71.11%)	71 (52.59%)	0.001
Blastocyst	79 (33.05%)	65 (28.89%)	64 (47.41%)	
Blastocysts with good quality n (%)				
0	19 (24.05%)	12 (18.46%)	6 (9.38%)	0.07
1	60 (75.95%)	53 (81.54%)	58 (90.62%)	
Cleavage embryos of good quality n (%)				
0	21 (13.13%)	18 (11.25%)	0 (0%)	0.003
1	68 (42.50%)	57 (35.63%)	23 (32.39%)	
2	71 (44.37%)	85 (53.12%)	48 (67.61%)	
β-HCG levels on the pregnancy day in women with clinical pregnancy median (IQR)				
Cleavage embyros transfer	616.50 (541.08)	748.10 (557.60)	928.80 (658.20)	0.013
Blastocyst transfer	1384.00 (1527.70)	1228.00 (865.20)	1541.50 (1044.75)	0.10

### MD between basal TSH and D14 TSH levels in pregnant and non-pregnant women

**Figure 1:**
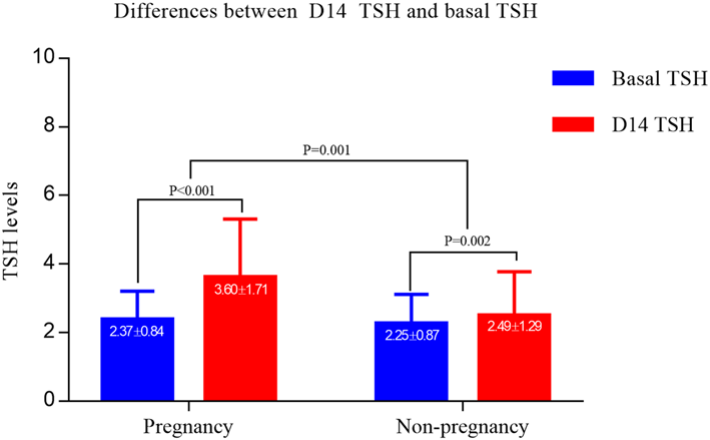
Mean difference between basal TSH levels and D14 TSH in pregnant and non-pregnant women. In both pregnant and non-pregnant women, D14 TSH levels were significantly elevated compared with basal TSH levels. The greater elevation of TSH levels was observed in pregnant women when compared with that in non-pregnant women.

In both pregnant and non-pregnant women, D14 TSH levels significantly rose compared to basal TSH levels (MD in pregnancy, 1.24 ± 1.51, *P* < 0.001; MD in non-pregnancy 0.24 ± 1.17, *P* = 0.002). However, the degree of TSH elevation was significantly higher in pregnant women compared to that in non-pregnant women (*P* = 0.001), as shown in Fig. 1.

### Primary outcomes in different D14 TSH groups

**Figure 2:**
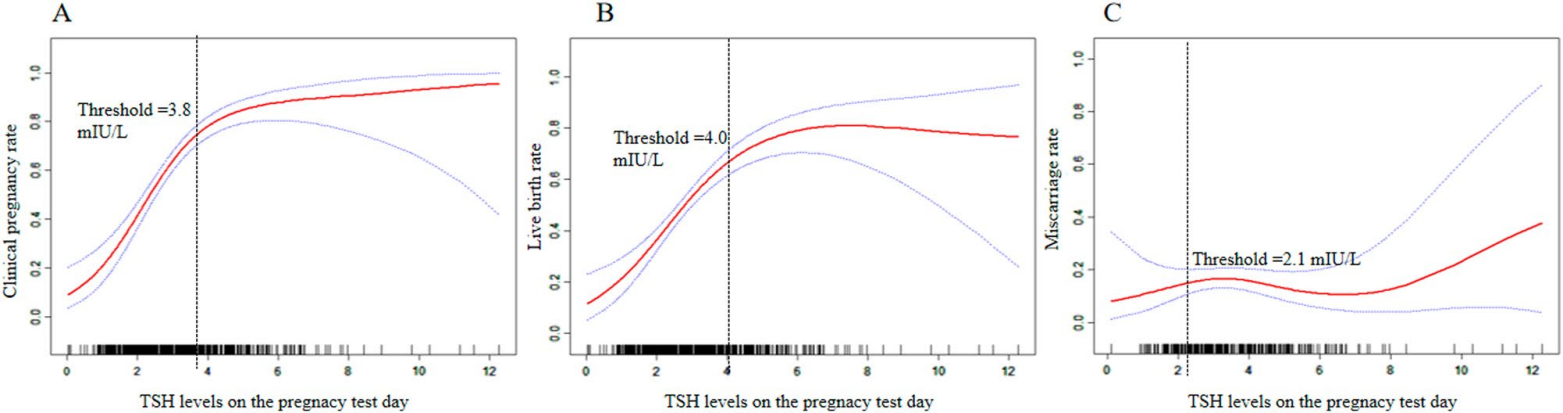
The relationship between the D14 TSH and the RR of primary outcomes analyzed by Generalized additive models with smoothing splines. (**A**) The RR of clinical pregnancy peaked at the threshold of 4.5 mIU/L and kept stable thereafter; (**B**) the RR of live birth peaked at the threshold of 5.5 mIU/L and decreased 30% when D14 TSH increased one unit. (**C**) The RR of miscarriage remained stable when the D14 TSH levels were less than 6.5 mIU/L and significantly increased 76% when D14 TSH increased one unit. RR: Relative risk; CP, Clinical pregnancy; LB, Live birth; Mis, Miscarriage; D14 TSH, Thyroid stimulating hormone levels 14 days after embryo transfer.

As shown in Table 2, the clinical pregnancy and live birth rates increased significantly in the high-normal D14 TSH group (67.11% vs. 42.67%, aRR = 3.39(2.22–5.19); 55.56% vs. 35.56%, aRR = 2.75(1.82–4.16), respectively), and doubled in the high D14 TSH group (83.70% vs. 42.67%, aRR = 8.00(4.35–14.73); 72.59% vs. 35.56%, aRR = 5.43(3.09–9.25), respectively) compared to the low TSH group. The miscarriage rates were similar among the different groups (*P* = 0.96). Furthermore, as shown in Fig. 2, when adjusted by age, AMH, E2, endometrial thickness, type and causes of infertility, and transferred embryos, the dose-dependent relationships between the D14 TSH and clinical pregnancy and live birth rates were observed. Specifically, the clinical pregnancy rate peaked at the threshold of 3.80 mIU/L [RR = 2.84(2.16, 3.73), *P* < 0.001], and kept stable thereafter [RR = 1.16(0.89, 1.51), *P* = 0.280]. Similarly, the live birth rate peaked at the threshold of 4.0 mIU/L [RR = 2.15(1.70, 2.71), *P* < 0.001] and kept stable thereafter [RR = 1.06(0.85, 1.32), *P* = 0.61]. The miscarriage rate remained stable within the whole D14 TSH range [RR = 1.03(0.84, 1.37), *P* = 0.78] (Table 3).

**Table 2:** Primary outcomes in different D14 TSH groups.

		Low (239)	High-normal (225)	High (135)	*P*
Clinical pregnancy	n(%)	102 (42.67%)^a^	151 (67.11%)^b^	113 (83.70%)^c^	< 0.001
	RR^d^	Ref	3.39 (2.22–5.19)	8.00 (4.35–14.73)	
Miscarriage	n(%)	14 (13.73%)	24 (15.89%)	15 (13.27%)	0.96
	RR^d^	Ref	1.32 (0.60–2.82)	1.09 (0.42–2.82)	
Live birth	n(%)	85 (35.56%)^a^	125 (55.56%)^b^	98 (72.59%)^b^	< 0.001
	RR^d^	Ref	2.75 (1.82–4.16)	5.43 (3.09–9.25)	

^a,^^b,c^Presence of different letters indicated that there were significant differences between the two groups.

^d^Adjusted by age, basal TSH, AMH, E2, endometrial thickness, type and causes of infertility, and transferred embryos.

**Table 3:** Obstetric outcomes in different D14 TSH groups.

	Low TSH (85)	High-normal TSH (125)	High TSH (98)	*P*
Singleton	76 (89.41%)	108 (86.40%)	80 (81.63%)	0.31
Twins	9 (10.59%)	17 (13.60%)	18 (18.37%)	
Cesarean delivery rate n(%)				
Singleton^a^	56 (73.68%)	70 (64.81%)	52 (65.82%)	0.41
Twins	9 (100.00%)	16 (94.12%)	17(94.44%)	0.69
Preterm birth rate n(%)				
Singleton^a^	6 (7.89%)	5 (4.62%)	7 (8.86%)	0.88
Twins	5 (55.56%)	10 (58.82%)	13 (72.22%)	0.61
Low birth weight rate n(%)				
Singleton^a^	6 (7.89%)	2 (1.85%)	3 (3.80%)	0.24
Twins	8 (44.44%)	14 (41.17%)	12 (33.33%)	0.68
Gestational age (weeks) mean ± SD				
Singleton^a^	38.95 ± 2.07	39.32 ± 1.32	38.91 ± 2.11	0.22
Twins	36.10 ± 1.94	36.77 ± 2.26	37.27 ± 1.22	0.29
Birth weight (g) mean ± SD				
Singleton^a^	3438.69 ± 642.69	3375.05 ± 442.37	3333.54 ± 567.24	0.48
Twins	2449.44 ± 566.08	2503.82 ± 483.77	2654.00 ± 382.92	0.23
Birth length (cm) mean ± SD				
Singleton^a^	50.18 ± 2.35	50.35 ± 1.16	50.14 ± 2.58	0.15
Twins	46.89 ± 3.88	47.27 ± 3.28	48.06 ± 2.93	0.39
Apgra score mean ± SD				
Singleton^a^	9.99 ± 0.12	9.98 ± 0.14	9.96 ± 0.25	0.64
Twins	9.78 ± 0.43	9.62 ± 1.13	9.92 ± 0.28	0.26

^a^Exclude the cycle with both one live birth and one still birth (n = 1).

### Secondary outcomes in different D14 TSH groups

Although there were similar proportions of singleton and twins in different D14 TSH groups as shown in Table 3, obstetric outcomes in singleton and twins live birth among the different D14 TSH groups were compared respectively. Additionally, very similar outcomes were observed in all the obstetric outcomes (All *P* > 0.05).

## Discussion

As far as we know, this is the largest prospective study investigating the association between D14 TSH levels and reproductive and obstetric outcomes in infertile women undergoing the first IVF/ICSI cycles. In this study, we demonstrated significantly higher D14 TSH levels and greater TSH elevation compared to basal TSH levels in pregnant women. The clinical pregnancy and live birth rates were significantly higher in the high D14 TSH group. Furthermore thresholds of 3.8 and 4.0 mIU/L were established to determine the change of possibility for clinical pregnancy and live birth rates respectively.

The impact of COH on thyroid had been extensively studied for decades. The first systematic review found doubtful effects of COH on the thyroid function due to the limited number and diverse general characteristics of included women^7^. However, the most recent meta-analysis performed by Busnelli et al. showed that TSH levels increased significantly in women undergoing IVF, and the TSH elevation was also observed on the pregnancy test day in pregnant women after ET^8^. However, Reinbllatt et al. included euthyroid patients who completed the ART cycles, and had estrogen and TSH levels measured at each visit during ART cycles^9^. They concluded that TSH levels remained relatively stable during the course of ART but increased significantly on the pregnancy test day in those who were pregnant. In addition to the similar result in terms of TSH increase on the pregnancy test day, we also claimed that TSH levels increased in women who did not conceive, and that the increase was significantly greater in pregnant women. The difference may be explained by the different ART protocols and sample size. In this prospective study, we included as far as we know the largest number of cycles using GnRH-a long protocol for ovarian stimulation, which made the results and conclusion more reliable. Another prospective study performed by Du et al. used the similar protocol as our study did and divided the patients into two group based on the basal TSH levels^10^. They demonstrated that D14 TSH levels were significantly higher than basal TSH levels, which was consistent with our study. It should be noted that although a total of 207 (137 in the low TSH group and 70 in the high-normal group) patients were included for analysis, only 126 patients underwent ET and had TSH levels measured 14 days or 28 days post ET. What’s more, the associations between TSH levels post ET and reproductive outcomes were not reported. As a result, it was hardly possible to determine the role and impact of D14 TSH on the reproductive outcomes.

Both Gracia et al. and Poppe et al. claimed that TSH levels peaked in 2 weeks post ET, and decreased thereafter^4,6^. During the whole trimesters, thyroid function in healthy pregnant women without structural thyroid disorders underwent dynamic changed to meet the increased demands of TH^15^, for which reason, population-based trimester-specified TSH or FT4 cutoff values were advised to be established^16^. When not available, the cutoff value of TSH for diagnosis of SCH was advised to be 4.0 mIU/L^17^. However, with the upper limit of TSH reference range for the general population as the cutoff value, a high proportion of 22.5% women in this study would be diagnosed as SCH due to the elevated D14 TSH levels.

Currently, it was well-known that β-HCG secreted by syncytiotrophoblast cells impacted thyroid gland by binding the TSH receptor, which led to increased TH levels and decreased TSH levels. Impaired thyroid response to β-HCG (which was defined as a high hCG with a high TSH or a low FT4 by the author) was reported to be associated with a lower crown rump length in the study performed by Zhang et al.^18^. However, it should be noted that the included pregnant women were at the median gestational age of 12.3 weeks, when the serum β-HCG levels were extremely high. However, in this prospective study, the included women (if pregnant) were at a gestation of 2 weeks, when the serum β-HCG levels were at the levels of 815.0 (515.4–1238.5) IU/L for cleavage embryo transfer and 1387.0 (825.2–2137.8) IU/L for blastocyst transfer. The results suggested that thyroid response to β-HCG at the gestational age of 2 weeks might be quite different from that in the late stage of first trimester and early stage of second trimester. In addition to the serum β-HCG levels, high estrogen levels were reported to impact the hypothalamic-pituitary-thyroid axis and alter the serum TH levels via the change of serum thyroid binding globulin^1^. In this prospective study, significantly higher estrogen levels were observed in the high D14 TSH groups compared to the low or high-normal D14 TSH groups, suggesting that high estrogen levels on the trigger day may associate with D14 TSH levels, which was consistent with Busnelli et al. in whose study a even longer impact of COH on thyroid function was claimed^19^.

SCH during pregnancy was associated with multiple adverse maternal and neonatal outcomes, and LT4 treatment was associated with improved clinical pregnancy outcome in SCH women undergoing ART^20–23^. Interestingly, without LT4 treatment for preconceptionally euthyroid women, we reported that D14 TSH levels were associated with clinical outcomes when using 4.2 mIU/L as the thresholds. In term of reproductive outcomes, we demonstrated that high D14 TSH group was associated with higher pregnancy and live birth rates but were not associated with obstetric outcomes. We further found the dose-dependent relationship between D14 TSH levels and the clinical outcomes using smoothing function, and identified the threshold values to predict the changes of possibility of clinical pregnancy rate, live birth rate. These results indicated that in preconceptionally euthyroid women who underwent COH, the clinical pregnancy and live birth rates significantly increased along with the elevation of D14 TSH levels, and reached the stable phase when D14 TSH levels passed the thresholds of 3.8 and 4.0 mIU/L respectively. Based on these observations, we would like to propose to screen thyroid function for the women who were biochemically pregnant on the pregnancy test day, and use 4.0 mIU/L as the threshold for medical intervention. However, the underlying mechanisms were largely unknown. One possible explanation was the interaction between TSH and leukaemia inhibitory factor (LIF)^24^. On the one hand, thyroid gland was reported to be a source of LIF production, and TSH influenced LIF secretion in cultured thyroid cells^25^. On the other hand, it was reported that LIF was involved in the early embryo implantation, while TSH could significantly stimulate the expression of LIF in endometrial cell cultures, which indicated the involvement of TSH in LIF signaling regulation and embryo implantation^26^.

There are some limitations of this study. (1) thyroid antibodies were missed. Most of the studies addressed the impaired reproductive outcomes in euthyroid women with thyroid antibodies^11,22,27,28^. In this prospective study, euthyroid women with both low-normal and high-normal TSH levels were included, which may lead to selection bias, and potentially limit the reliability of the results. However, we also believed that not restricting the population based on the presence of thyroid antibodies may generalize the results to more patients. (2) Serum estrogen and TBG levels were missed. After oocytes pick-up, the estrogen levels decreased significantly. TBG levels were supposed to changed accordingly. However, when the women were pregnant post ET, the estrogen and TBG levels changed again as a response to pregnancy. Without these data, whether and how they impaced t D14 TSH levels could not be fully understood. (3) Although the sample size was relatively large, we did not perform the sample size calculation. As a consequence, the power of this study was partially reduced. Besides, due to the limited number of women transferred with cleavage embryo or D5 blastocyst, further subgroup analyses based on the stage of transferred embryos seemed unreliable. (4) We included women using GnRH-a ovarian stimulation protocol, and the general patients’ characteristics and estrogen levels may be significantly different from those with mild ovarian stimulation protocols. As a consequence, the results might only be applied to the fixed population. (5) Although we demonstrated the associations between D14 TSH levels and clinical pregnancy rate and live birth rate, the causal relationships could not be drawn. Whether treatment could improve the relative low clinical pregnancy and live birth rates when the D14 TSH levels were less than the threshold should further be investigated.

The strengths of this study are listed as follows: (1) the included patients were as homogeneous as possible due to the uniform COS protocol, which added reliability to the study. (2) The linear associations between D14 TSH levels and above-mentioned reproductive outcomes were firstly reported, and the results suggested that using a uniform reference range (4.0 mIU/L) for the assessment of thyroid function was appropriate. (3) the results also for the first time showed little impact of high D14 TSH levels on the obstetric outcomes, which suggested that D14 TSH levels would soon declined into the normal trimester-specific ranges.

## Conclusion

Elevated D14 TSH levels were associated with better clinical pregnancy and live birth rates, and not associated with worse obstetric outcomes. The mechanisms to explain the phenomenon remained to be studied.

## Data Availability

The data used during the current study are available from the corresponding author on a reasonable request.
